# One mol­ecule, three crystal structures: conformational trimorphism of *N*-[(1*S*)-1-phenyl­eth­yl]benzamide

**DOI:** 10.1107/S2056989020008877

**Published:** 2020-07-07

**Authors:** Fermin Flores Manuel, Martha Sosa Rivadeneyra, Sylvain Bernès

**Affiliations:** a Facultad de Ciencias Químicas, Benemérita Universidad Autónoma de Puebla, 72570 Puebla, Pue., Mexico; b Instituto de Física, Benemérita Universidad Autónoma de Puebla, 72570 Puebla, Pue., Mexico

**Keywords:** crystal structure, conformational polymorphism, benzamide, hydrogen bond, chain motif

## Abstract

The title compound, C_15_H_15_NO, is an enanti­opure small mol­ecule, which has been synthesized many times, although its crystal structure was never determined. By recrystallization from a variety of solvent mixtures (pure aceto­nitrile, ethanol–water, toluene–ethanol, THF–methanol), we obtained three unsolvated polymorphs, in space groups *P*2_1_ and *P*2_1_2_1_2_1_. Form **I** is obtained from aceto­nitrile, without admixture of other forms, whereas forms **II** and **III** are obtained simultaneously by concomitant crystallizations from alcohol-based solvent mixtures. All forms share the same supra­molecular structure, based on infinite *C*_1_^1^(4) chain motifs formed by N—H⋯O inter­molecular hydrogen bonds, as usual for non-sterically hindered amides. However, a conformational modification of the mol­ecular structure, related to the rotation of the phenyl rings, alters the packing of the chains in the crystal structures. The orientation of the chain axis is perpendicular and parallel to the crystallographic twofold screw axis of space group *P*2_1_ in forms **I** and **II**, respectively. As for form **III**, the asymmetric unit contains two independent mol­ecules forming parallel chains in space group *P*2_1_2_1_2_1_, and the crystal structure combines features of monoclinic forms **I** and **II**.

## Abstract 2

The conformational trimorphism of a chiral amide is described, in space groups *P*2_1_ and *P*2_1_2_1_2_1_, with different orientations of the supra­molecular one-dimensional structures with respect to the twofold screw axis.

## Chemical context   

The study of polymorphism is paramount in the field of organic materials, especially in the design of new active pharmaceutical ingredients, either for tailoring their bioavailability, or for legal reasons related to patent rights and intellectual property. Walter McCrone (1965 ▸) famously stated more than 50 years ago that ‘the number of [polymorphic] forms known for a given compound is proportional to the time and money spent in research on that compound’. Today, it seems that this statement still holds true, and that a large proportion of the discovered polymorphs are obtained in a non-planned way. In the current situation, the rules allowing (or avoiding) a mol­ecular system to crystallize with several forms are not fully understood, although assessing the risk of polymorphism is workable to some extent. For example, the CSD-Materials module available in *Mercury* can perform predictions on a polymorphic target compound, through an estimation of its hydrogen-bonding landscape (Feeder *et al.*, 2015 ▸; Macrae *et al.*, 2020 ▸).

A recent survey of the CSD (Groom *et al.*, 2016 ▸) showed that polymorphism prevalence among single-component organic anhydrates constitutes about 1.22% of compounds for which at least one crystal structure is known (Kersten *et al.*, 2018 ▸). A similar figure was obtained using the Merck index as a source of data: for 10330 compounds present in the 12th edition (1996), 1.4% were polymorphic (Stahly, 2007 ▸).

However, compounds appearing only once in the CSD might exist in other polymorphic forms that have still not been crystallized. It also seems hard to believe that all mol­ecules should be necessarily polymorphous, as sometimes claimed. For example, huge amounts of ibuprofen [2-(4-iso­butyl­phen­yl)propanoic acid] have been produced since its introduction as a painkiller in 1969. Notwithstanding the numerous studies carried out on this small mol­ecule, only one crystalline form is known. But there is no doubt that from a statistical point of view, trimorphic systems are much less common than dimorphic systems, tetra­morphic systems are in turn much less common than trimorphic systems, *etc*. A rule of thumb is that a tenfold drop is observed for the prevalence of *n*-morphism in comparison to (*n*-1)-morphism (*n* ≥ 3). It is not surprising that, for example, well-characterized hexa­morphism is exceptional (Yu *et al.*, 2000 ▸). Another empirical observation is that more polymorphs are reported for small mol­ecules (less than 30 C atoms per formula) compared to large ones, because of the correlation between mol­ecular complexity and the difficulty of synthesizing large mol­ecules. These observations are in line with McCrone’s statement, and today there is a consensus that polymorphism is a pervasive phenomenon, which occurs on a random basis and remains poorly predictable (Cruz-Cabeza *et al.*, 2015 ▸).

Within this context, we report a case of trimorphism, for a low-mol­ecular-weight chiral mol­ecule, for which the crystal structure was never established, even though many researchers have used it as a reagent since its first reported synthesis (Bezruchko *et al.*, 1967 ▸).
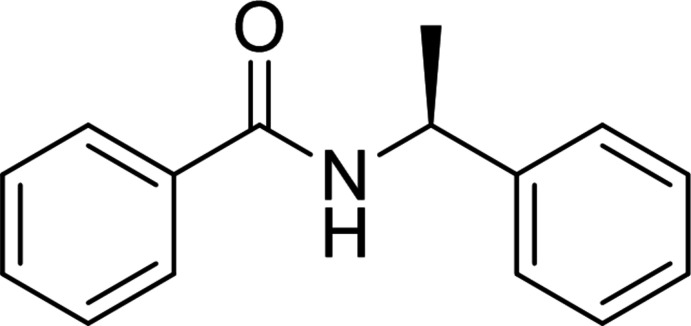


## Mol­ecular and crystal structures   

We used *N*-[(1*S*)-1-phenyl­eth­yl]benzamide as a component for co-crystallization with other small mol­ecules having a high hydrogen-bond propensity. While probing a variety of solvents for the crystallization of the free amide, we recovered three non-solvated polymorphs, in a reproducible manner. Form **I** (*P*2_1_) was obtained from aceto­nitrile, and its measured melting point and angle of optical rotation match data reported by other groups (*e.g*. Karnik & Kamath, 2008 ▸). Forms **II** (*P*2_1_) and **III** (*P*2_1_2_1_2_1_) were obtained as concomitant crystals, by using ethanol–water, toluene–ethanol or THF–methanol mixtures. Simulated X-ray powder patterns are clearly different for each form, confirming that true polymorphs were crystallized.

**Figure 1:**
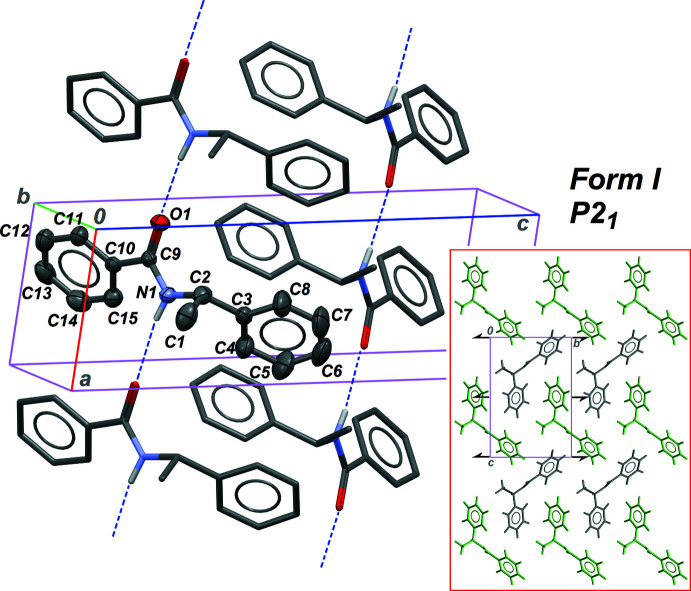
Part of the crystal structure of form **I**, with the asymmetric unit displayed with displacement ellipsoids for non-H atoms at the 30% probability level. C-bound H atoms are omitted for clarity, and hydrogen bonds forming the infinite 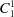
(4) chains are drawn as dashed lines. The inset is the crystal structure viewed down the chain axis, parallel to the crystallographic *a* axis. Grey and green mol­ecules are related by the 2_1_ symmetry elements parallel to [010] in space group *P*2_1_.

Forms **I** and **II** share the same crystal symmetry (Table 5 ▸), but have very different densities, 1.157 and 1.208 g cm^−3^, respectively. It can therefore be predicted that mol­ecules are packed in the solid state in a more efficient manner for **II**, compared to **I**. However, both forms display the same supra­molecular structure, based on the classical 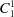
(4) chain motif, which is the most common for amide derivatives (Figs. 1 ▸ and 2 ▸). The N—H⋯O hydrogen bond is stronger for **I**, while an opposite situation should be expected if one considers crystal densities (Tables 1 ▸ and 2 ▸). The factor triggering polymorphism is, in this case, related to the mol­ecular structure. The conformation of the mol­ecule is modified by rotation of the phenyl ring C3–C8 bonded to the chiral centre, while the position of the other peripheral phenyl group, C10–C15, remains almost unchanged with respect to the amide group. Dihedral angles involved in the mol­ecular conformation are given in Table 4 ▸: angle N1—C9—C10—C15 is modified by *ca* 3° between the two forms, while the other angle, N1—C2—C3—C4, is modified by *ca* 14°. As a consequence, the dihedral angle between the phenyl rings is 23.1 (2) and 56.2 (1)° in **I** and **II**, respectively.

**Figure 2:**
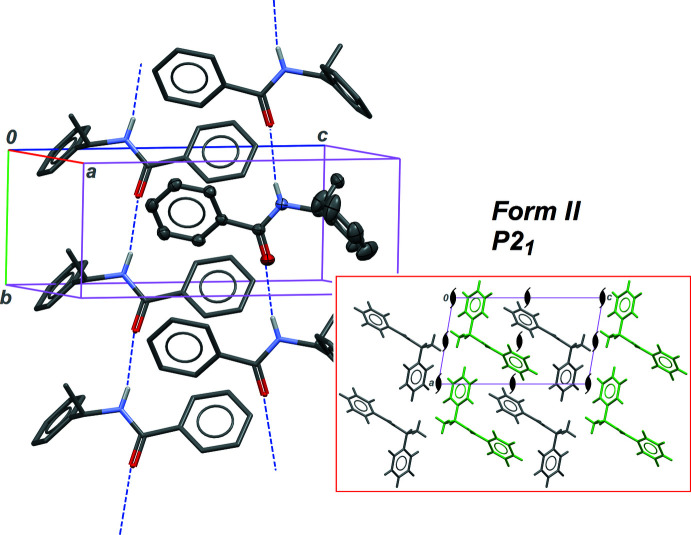
Part of the crystal structure of form **II**, using the same style as for Fig. 1 ▸. The labelling scheme is as in **I**. In the inset, the projection axis is [010].

The conformational modification leads to different arrangements for the infinite *C*(4) chains in the crystals. In **I**, the 1D motif is running in the [100] direction, and is thus normal to the twofold screw axis (Fig. 1 ▸, inset). The 2_1_ symmetry element relates neighbouring chains in the crystal, resulting in a relative orientation of the chains that is unfavourable for the packing of the phenyl rings: inter-chain dihedral angles between phenyl groups are close to 90°: δ_1→1′_ = 88.4 (3)°, δ_1→2′_ = 84.9 (2)° and δ_2→2′_ = 70.8 (2)°, where 1 and 2 stand for rings C3–C8 and C10–C15, while a primed ring is related to a non-primed ring through the symmetry element 2_1_. These angles were calculated using *PLATON* (Spek, 2020 ▸), and only non-parallel rings are considered. In contrast, the crystal structure of form **II** is built on *C*(4) chains parallel to the screw axis, in the [010] direction. As in the previous case, two neighbouring chains are related through the 2_1_ axis. However, given that chains and symmetry elements share the same direction, some inter-chain inter­actions feature phenyl rings in a less perpendicular arrangement: δ_1→1′_ = 85.2 (2)°, δ_1→2′_ = 80.3 (2)°, δ_2→1′_ = 56.2 (2)° and δ_2→2′_ = 64.7 (1)°. Chains are then more densely packed, to afford a material with higher density (Fig. 2 ▸, inset). These different packing structures, in the same space group, are also reflected in different Kitaigorodskii packing index: 0.638 for **I** and 0.670 for **II** (Spek, 2020 ▸).

**Figure 3:**
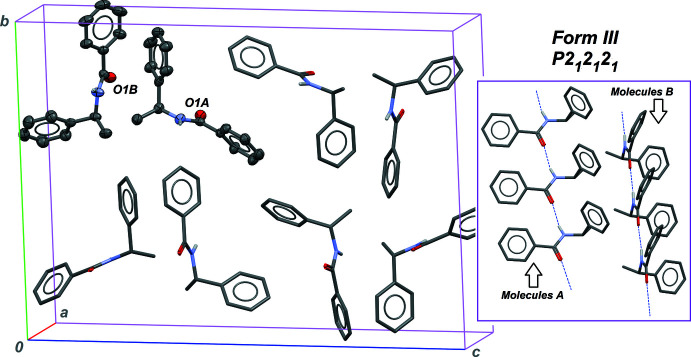
Part of the crystal structure of form **III**. Left: unit-cell content is represented, as in Figs. 1 ▸ and 2 ▸. The labelling scheme is identical, with *A* and *B* suffixes for the two independent mol­ecules. Right: two neighbouring 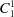
(4) chains based on independent mol­ecules *A* and *B* are represented.

The third polymorph, **III**, includes two independent mol­ecules in the asymmetric unit of an ortho­rhom­bic cell, each one forming a supra­molecular structure identical to those of forms **I** and **II** [infinite 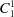
(4) chains parallel to the *a* axis for mol­ecules *A* and *B*, see Table 3 ▸ and Fig. 3 ▸]. The mol­ecular conformation is similar for *A* and *B* mol­ecules, and can be described as inter­mediary between conformations stabilized in crystals **I** and **II**: the phenyl ring bonded to the chiral C atom is configured as in crystal **II**, while the other phenyl group is oriented as in crystal **I** (Table 4 ▸). The intra­molecular dihedral angle between phenyl rings is therefore also midway: 47.0 (1)° for mol­ecules *A* and 47.4 (1)° for mol­ecules *B*.

**Figure 4:**
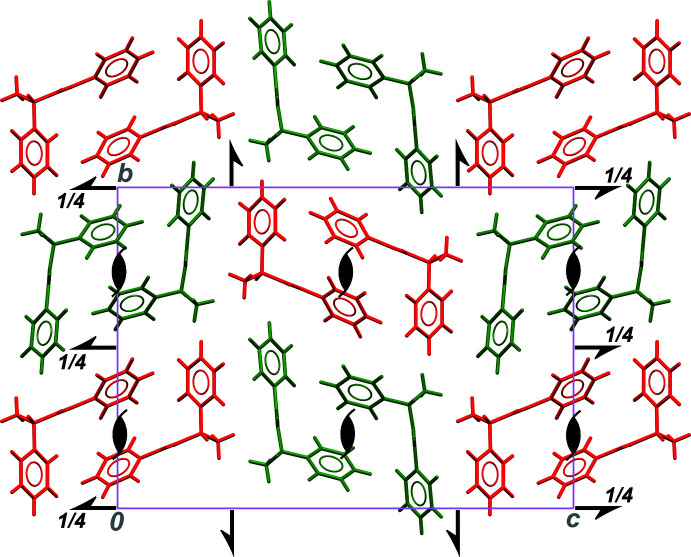
Part of the crystal structure of form **III**, viewed down the chain axis, parallel to the crystallographic *a* axis. Red and green mol­ecules belong to the *A* and *B* families, respectively. All symmetry elements of space group *P*2_1_2_1_2_1_ are positioned.

With such a configuration, it is not surprising to obtain a crystal structure for **III** in space group *P*2_1_2_1_2_1_ simultaneously reminiscent of those observed for **I** and **II** (Fig. 4 ▸). The twofold screw axis parallel to [100] gives an arrangement similar to that described in form **II**, with two neighbouring *C*(4) chains including mol­ecules from the same family, *A*/*A* or *B*/*B*, closely packed around this symmetry element. On the other hand, the packing in directions perpendicular to the chain axis is based on screw axes along [010] and [001], and is thus similar to that observed in form **I** with regard to neighbouring crystallographically independent mol­ecules, *A*/*B* or *B*/*A*. The ortho­rhom­bic form **III** with *Z*′ = 2 can be seen as a mixture combining features of *Z*′ = 1 monoclinic forms **I** and **II**. This is consistent with metrics directly related to packing efficiency, which fall between those of phases **I** and **II**: the calculated density for **III** is 1.199 g cm^−3^, the Kitaigorodskii packing index is 0.666, and large inter­molecular dihedral angles δ_*p*→*q*′_ between phenyl rings in neighbouring chains are in the range 70.1 (2) to 89.7 (2)°.

## Database survey   

From the previous description, it is clear that the conformational trimorphism for the title compound is a consequence of the rotation of the peripheral phenyl rings, which changes their environment, affecting the packing of the *C*(4) chains. This mol­ecular flexibility is confirmed by the crystal-structure determination of the unique co-crystal reported to date including the title mol­ecule (Tinsley *et al.*, 2017 ▸): the conformation is far from that observed in the free amide we report, and one phenyl is even disordered by rotation.

Polymorphism can then occur, although the hydrogen-bonded pattern remains unaltered. Such a behaviour has been invoked to rationalize the crystallization of the highly metastable ortho­rhom­bic form of benzamide, for which the space group is still controversial (*Pba2*: Blagden *et al.*, 2005 ▸; *Fdd*2: Johansson & van de Streek, 2016 ▸). In the same way, the twisting between the nitro­phenyl and the thio­phene rings in the pharmaceutical inter­mediate 5-methyl-2-[(2-nitro­phen­yl)amino]-3-thio­phene­carbo­nitrile is related to the rich polymorphism of this compound: six forms have been structurally characterized in this case, with a variety of colours and shapes (Yu *et al.*, 2000 ▸; Price *et al.*, 2005 ▸).

Regarding the supra­molecular structure observed in the title compound, the imposed supra­molecular motif limits the scope for polymorphism. Indeed, the frequency of infinite chains in the crystal structures of amides is as high as 28.2% in the CSD (version 5.41, updated May 2020; both organic and metal–organic amides were considered), and is probably higher for non-sterically hindered amides, such as the title compound. Moreover, the title amide having only one donor and one acceptor sites, any variation of the supra­molecular structure is very unlikely. However, it should be noted that this 1D structure is easily propagated through a screw axis in the crystal state. A survey of the organic amides crystallizing in Sohncke (*i.e.* non-enanti­ogenic) space groups reveals that for 449 hits, 83% are reported in space groups *P*2_1_ and *P*2_1_2_1_2_1_, while the combined frequency of these groups over the whole CSD database is only 12%. It thus seems that any space group including rototranslations can fit a polymorphic form of a small amide, either enanti­opure or achiral, regardless of the rigidity of the supra­molecular structure. We could anti­cipate that crystallization of other forms of the title compound could be achieved, for example, in space groups *P*2_1_2_1_2, or *P*3_1_, among others.

## Synthesis and crystallization   

The title compound was synthesized using a literature method (Tang, 2005 ▸). A solution of benzoic acid was prepared (1 g, 8.18 mmol in 50 mL toluene), and 50 mg of boric acid, B(OH)_3_, was added, followed by (*S*)-1-phenyl­ethyl­amine (0.9 g, 7.44 mmol). This mixture was refluxed for 36 h, after which the reaction was complete (TLC, SiO_2_, hexa­ne:AcOEt 1:1). After cooling to room temperature, 200 mL of hexane were added, affording the title compound as a white precipitate, which was separated. Yield, 90%. Single crystals were obtained by slow evaporation of solutions (0.01 g in 10 mL): with aceto­nitrile at 298 K, pure form **I** was recrystallized; [α]_D_ −17.1 (*c* 1, CHCl_3_), m.p. 395 K [literature: −17.9 (*c* 1, CHCl_3_), 395–396 K; Karnik & Kamath, 2008 ▸]. Concomitant crystallizations of forms **II** and **III** were realized at 298 K in ethanol–water (97:3 *v*/*v*) or ethanol–toluene (1:1, *v*/*v*). Given that all of the crystals are colourless and prism-shaped, the crystal form can not be assigned visually.

## Refinement   

Crystal data, data collection and structure refinement details are summarized in Table 5 ▸. C-bound hydrogen atoms were placed in calculated positions and refined using a riding model with *U*_iso_(H) = 1.2*U*_eq_(C), C—H = 0.93 Å, and C—H = 0.96 Å, *U*_iso_(H) = 1.5*U*_eq_(C), for aromatic and methyl hydrogen atoms, respectively. Amide hydrogen atoms (H1 in **I** and **II;** H1*A* and H1*B* in **III**) were found in difference maps and their coordinates were freely refined with *U*_iso_(H) = 1.2*U*_eq_(N).

## Supplementary Material

Crystal structure: contains datablock(s) I, II, III, global. DOI: 10.1107/S2056989020008877/yk2134sup1.cif

Structure factors: contains datablock(s) I. DOI: 10.1107/S2056989020008877/yk2134Isup2.hkl

Structure factors: contains datablock(s) II. DOI: 10.1107/S2056989020008877/yk2134IIsup3.hkl

Structure factors: contains datablock(s) III. DOI: 10.1107/S2056989020008877/yk2134IIIsup4.hkl

Supporting information file. DOI: 10.1107/S2056989020008877/yk2134Isup5.cml

CCDC references: 2013243, 2013244, 2013245

Additional supporting information:  crystallographic information; 3D view; checkCIF report

## Figures and Tables

**Table 1:** Hydrogen-bond geometry (Å, °) for form **I** [Chem scheme1]

*D*—H⋯*A*	*D*—H	H⋯*A*	*D*⋯*A*	*D*—H⋯*A*
N1—H1⋯O1^i^	0.85 (3)	2.11 (3)	2.952 (3)	169 (3)

Symmetry code: (i) 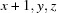.

**Table 2:** Hydrogen-bond geometry (Å, °) for form **II** [Chem scheme1]

*D*—H⋯*A*	*D*—H	H⋯*A*	*D*⋯*A*	*D*—H⋯*A*
N1—H1⋯O1^i^	0.84 (3)	2.30 (3)	3.123 (2)	168 (2)

Symmetry code: (i) 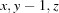.

**Table 3:** Hydrogen-bond geometry (Å, °) for form **III** [Chem scheme1]

*D*—H⋯*A*	*D*—H	H⋯*A*	*D*⋯*A*	*D*—H⋯*A*
N1*A*—H1*A*⋯O1*A* ^i^	0.87 (3)	2.23 (3)	3.080 (3)	167 (3)
N1*B*—H1*B*⋯O1*B* ^i^	0.85 (3)	2.22 (3)	3.047 (3)	163 (3)

Symmetry code: (i) 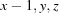.

**Table 4:** Intra­molecular dihedral angles describing the conformations of the title compound in forms **I**, **II** and **III**

Dihedral angle (°)	*P*2_1_ form **I**	*P*2_1_ form **II**	*P*2_1_2_1_2_1_ form **III** ^*a*^
N1—C2—C3—C4	−27.3 (5)	−41.1 (3)	−42.5 (4), −44.4 (4)
N1—C9—C10—C15	−34.2 (4)	−31.2 (3)	−37.9 (4), −36.6 (4)
Phen­yl⋯phen­yl	23.1 (2)	56.2 (1)	47.0 (1), 47.4 (1)

Note: (*a*) *Z*′ = 2.

**Table 5:** Experimental details

	Form **I**	Form **II**	Form **III**
Crystal data			
Chemical formula	C_15_H_15_NO	C_15_H_15_NO	C_15_H_15_NO
*M*_r_	225.28	225.28	225.28
Crystal system, space group	Monoclinic, *P*2_1_	Monoclinic, *P*2_1_	Orthorhombic, *P*2_1_2_1_2_1_
Temperature (K)	295	295	295
*a*, *b*, *c* (Å)	5.0472 (4), 9.3118 (7), 13.9581 (15)	8.3496 (6), 5.2632 (2), 14.2969 (10)	5.2133 (3), 18.3625 (12), 26.0799 (19)
α, β, γ (°)	90, 99.708 (8), 90	90, 99.800 (6), 90	90, 90, 90
*V* (Å^3^)	646.62 (10)	619.12 (7)	2496.6 (3)
*Z*	2	2	8
Radiation type	Ag *K*α, λ = 0.56083 Å	Ag *K*α, λ = 0.56083 Å	Ag *K*α, λ = 0.56083 Å
μ (mm^−1^)	0.05	0.05	0.05
Crystal size (mm)	0.37 × 0.12 × 0.09	0.34 × 0.19 × 0.14	0.27 × 0.25 × 0.08

Data collection			
Diffractometer	Stoe Stadivari	Stoe Stadivari	Stoe Stadivari
Absorption correction	Multi-scan (*X-AREA*; Stoe & Cie, 2019 ▸)	Multi-scan (*X-AREA*; Stoe & Cie, 2019 ▸)	Multi-scan (*X-AREA*; Stoe & Cie, 2019 ▸)
*T*_min_, *T*_max_	0.313, 1.000	0.574, 1.000	0.361, 1.000
No. of measured, independent and observed [*I* > 2σ(*I*)] reflections	15392, 2833, 1573	14738, 2456, 1847	46311, 5439, 2319
*R*_int_	0.066	0.037	0.132
(sin θ/λ)_max_ (Å^−1^)	0.639	0.639	0.639

Refinement			
*R*[*F* ^2^ > 2σ(*F* ^2^)], *wR*(*F* ^2^), *S*	0.043, 0.102, 0.84	0.036, 0.090, 0.94	0.038, 0.080, 0.74
No. of reflections	2833	2456	5439
No. of parameters	159	159	316
No. of restraints	1	1	0
H-atom treatment	H atoms treated by a mixture of independent and constrained refinement	H atoms treated by a mixture of independent and constrained refinement	H atoms treated by a mixture of independent and constrained refinement
Δρ_max_, Δρ_min_ (e Å^−3^)	0.11, −0.12	0.12, −0.14	0.11, −0.10

Computer programs: *X-AREA* and *X-RED32* (Stoe & Cie, 2019 ▸), *SHELXT2018/2* (Sheldrick, 2015*a* ▸), *SHELXL2018/3* (Sheldrick, 2015*b* ▸), *Mercury* (Macrae *et al.*, 2020 ▸) and *publCIF* (Westrip, 2010 ▸).

## supplementary crystallographic information

### N-[(1S)-1-Phenylethyl]benzamide (I) Crystal data

**Table:**

C_15_H_15_NO	*D*_x_ = 1.157 Mg m^−^^3^
*M**_r_* = 225.28	Melting point: 395 K
Monoclinic, *P*2_1_	Ag *K*α radiation, λ = 0.56083 Å
*a* = 5.0472 (4) Å	Cell parameters from 7505 reflections
*b* = 9.3118 (7) Å	θ = 2.9–21.0°
*c* = 13.9581 (15) Å	µ = 0.05 mm^−^^1^
β = 99.708 (8)°	*T* = 295 K
*V* = 646.62 (10) Å^3^	Prism, colourless
*Z* = 2	0.37 × 0.12 × 0.09 mm
*F*(000) = 240	

### N-[(1S)-1-Phenylethyl]benzamide (I) Data collection

**Table:**

Stoe Stadivari diffractometer	2833 independent reflections
Radiation source: Sealed X-ray tube, Axo Astix-f Microfocus source	1573 reflections with *I* > 2σ(*I*)
Graded multilayer mirror monochromator	*R*_int_ = 0.066
Detector resolution: 5.81 pixels mm^-1^	θ_max_ = 21.0°, θ_min_ = 2.9°
ω scans	*h* = −5→6
Absorption correction: multi-scan (X-AREA; Stoe & Cie, 2019)	*k* = −11→11
*T*_min_ = 0.313, *T*_max_ = 1.000	*l* = −17→17
15392 measured reflections	

### N-[(1S)-1-Phenylethyl]benzamide (I) Refinement

**Table:**

Refinement on *F*^2^	Secondary atom site location: difference Fourier map
Least-squares matrix: full	Hydrogen site location: mixed
*R*[*F*^2^ > 2σ(*F*^2^)] = 0.043	H atoms treated by a mixture of independent and constrained refinement
*wR*(*F*^2^) = 0.102	*w* = 1/[σ^2^(*F*_o_^2^) + (0.0482*P*)^2^] where *P* = (*F*_o_^2^ + 2*F*_c_^2^)/3
*S* = 0.84	(Δ/σ)_max_ < 0.001
2833 reflections	Δρ_max_ = 0.11 e Å^−^^3^
159 parameters	Δρ_min_ = −0.12 e Å^−^^3^
1 restraint	Extinction correction: SHELXL-2018/3 (Sheldrick, 2015b), Fc^*^=kFc[1+0.001xFc^2^λ^3^/sin(2θ)]^-1/4^
0 constraints	Extinction coefficient: 0.110 (15)
Primary atom site location: dual	

### N-[(1S)-1-Phenylethyl]benzamide (I) Fractional atomic coordinates and isotropic or equivalent isotropic displacement parameters (Å^2^)

**Table:**

	*x*	*y*	*z*	*U*_iso_*/*U*_eq_	
O1	0.0514 (3)	0.4480 (2)	0.20994 (15)	0.0634 (7)	
N1	0.4967 (5)	0.4138 (3)	0.24782 (19)	0.0552 (7)	
H1	0.648 (6)	0.434 (4)	0.232 (2)	0.066*	
C1	0.5907 (9)	0.1568 (5)	0.2590 (3)	0.0930 (13)	
H1A	0.775962	0.172744	0.254262	0.140*	
H1B	0.488542	0.143158	0.195139	0.140*	
H1C	0.575931	0.072839	0.297644	0.140*	
C2	0.4818 (6)	0.2871 (4)	0.3067 (2)	0.0644 (10)	
H2	0.291287	0.268881	0.308304	0.077*	
C3	0.6215 (6)	0.3062 (4)	0.4109 (2)	0.0607 (9)	
C4	0.8300 (8)	0.3981 (5)	0.4371 (3)	0.0897 (13)	
H4	0.889422	0.454313	0.389872	0.108*	
C5	0.9556 (10)	0.4096 (7)	0.5331 (3)	0.1221 (18)	
H5	1.099463	0.472265	0.549813	0.147*	
C6	0.8674 (14)	0.3286 (9)	0.6029 (4)	0.125 (2)	
H6	0.946930	0.337460	0.667740	0.150*	
C7	0.6669 (16)	0.2373 (8)	0.5769 (4)	0.143 (2)	
H7	0.609507	0.180178	0.624109	0.171*	
C8	0.5405 (10)	0.2247 (6)	0.4809 (3)	0.1114 (17)	
H8	0.399651	0.160002	0.464684	0.134*	
C9	0.2809 (5)	0.4846 (3)	0.20237 (19)	0.0465 (7)	
C10	0.3311 (5)	0.6101 (3)	0.1421 (2)	0.0476 (8)	
C11	0.1515 (6)	0.6394 (4)	0.0594 (2)	0.0674 (10)	
H11	0.000363	0.581618	0.042805	0.081*	
C12	0.1920 (9)	0.7537 (5)	0.0002 (3)	0.0898 (14)	
H12	0.072176	0.770246	−0.057018	0.108*	
C13	0.4068 (10)	0.8418 (5)	0.0258 (4)	0.0922 (15)	
H13	0.431123	0.920152	−0.013150	0.111*	
C14	0.5853 (8)	0.8160 (4)	0.1077 (4)	0.0855 (13)	
H14	0.731981	0.876693	0.124659	0.103*	
C15	0.5512 (6)	0.6993 (4)	0.1667 (3)	0.0646 (10)	
H15	0.675967	0.681326	0.222472	0.078*	

### N-[(1S)-1-Phenylethyl]benzamide (I) Atomic displacement parameters (Å^2^)

**Table:**

	*U*^11^	*U*^22^	*U*^33^	*U*^12^	*U*^13^	*U*^23^
O1	0.0432 (9)	0.0760 (17)	0.0717 (14)	−0.0023 (11)	0.0114 (9)	0.0127 (13)
N1	0.0412 (12)	0.0636 (18)	0.0604 (15)	−0.0008 (13)	0.0074 (11)	0.0189 (14)
C1	0.130 (3)	0.060 (3)	0.081 (3)	0.000 (2)	−0.006 (2)	0.007 (2)
C2	0.0567 (17)	0.061 (2)	0.074 (2)	−0.0070 (17)	0.0069 (17)	0.020 (2)
C3	0.0590 (17)	0.067 (2)	0.058 (2)	0.0136 (18)	0.0154 (16)	0.0182 (19)
C4	0.095 (3)	0.113 (4)	0.057 (2)	−0.011 (3)	0.0018 (19)	0.008 (2)
C5	0.136 (4)	0.146 (5)	0.073 (3)	−0.007 (4)	−0.015 (3)	−0.013 (4)
C6	0.148 (5)	0.162 (6)	0.057 (3)	0.064 (4)	−0.006 (3)	0.009 (4)
C7	0.177 (6)	0.178 (7)	0.075 (4)	0.021 (5)	0.032 (4)	0.061 (4)
C8	0.119 (3)	0.137 (4)	0.078 (3)	−0.013 (3)	0.015 (3)	0.049 (3)
C9	0.0449 (15)	0.054 (2)	0.0411 (16)	0.0003 (14)	0.0097 (12)	−0.0029 (15)
C10	0.0457 (14)	0.051 (2)	0.0483 (17)	0.0075 (14)	0.0149 (13)	0.0019 (15)
C11	0.0660 (18)	0.077 (3)	0.059 (2)	0.0056 (19)	0.0087 (16)	0.013 (2)
C12	0.095 (3)	0.104 (4)	0.070 (3)	0.021 (3)	0.016 (2)	0.031 (3)
C13	0.101 (3)	0.078 (3)	0.108 (4)	0.023 (3)	0.050 (3)	0.044 (3)
C14	0.081 (2)	0.060 (3)	0.121 (4)	−0.006 (2)	0.033 (3)	0.015 (3)
C15	0.064 (2)	0.060 (2)	0.069 (2)	0.0007 (17)	0.0097 (17)	0.0092 (19)

### N-[(1S)-1-Phenylethyl]benzamide (I) Geometric parameters (Å, º)

**Table:**

O1—C9	1.229 (3)	C6—H6	0.9300
N1—C9	1.338 (3)	C7—C8	1.388 (8)
N1—C2	1.448 (4)	C7—H7	0.9300
N1—H1	0.85 (3)	C8—H8	0.9300
C1—C2	1.530 (5)	C9—C10	1.486 (4)
C1—H1A	0.9600	C10—C11	1.370 (4)
C1—H1B	0.9600	C10—C15	1.383 (4)
C1—H1C	0.9600	C11—C12	1.384 (5)
C2—C3	1.515 (5)	C11—H11	0.9300
C2—H2	0.9800	C12—C13	1.358 (6)
C3—C8	1.354 (5)	C12—H12	0.9300
C3—C4	1.358 (5)	C13—C14	1.352 (6)
C4—C5	1.386 (5)	C13—H13	0.9300
C4—H4	0.9300	C14—C15	1.392 (5)
C5—C6	1.365 (8)	C14—H14	0.9300
C5—H5	0.9300	C15—H15	0.9300
C6—C7	1.325 (8)		
			
C9—N1—C2	123.7 (2)	C6—C7—C8	121.6 (5)
C9—N1—H1	118 (2)	C6—C7—H7	119.2
C2—N1—H1	118 (2)	C8—C7—H7	119.2
C2—C1—H1A	109.5	C3—C8—C7	120.2 (5)
C2—C1—H1B	109.5	C3—C8—H8	119.9
H1A—C1—H1B	109.5	C7—C8—H8	119.9
C2—C1—H1C	109.5	O1—C9—N1	121.7 (3)
H1A—C1—H1C	109.5	O1—C9—C10	121.3 (3)
H1B—C1—H1C	109.5	N1—C9—C10	116.9 (2)
N1—C2—C3	112.9 (3)	C11—C10—C15	118.7 (3)
N1—C2—C1	110.0 (3)	C11—C10—C9	118.8 (3)
C3—C2—C1	111.6 (3)	C15—C10—C9	122.6 (3)
N1—C2—H2	107.4	C10—C11—C12	120.8 (4)
C3—C2—H2	107.4	C10—C11—H11	119.6
C1—C2—H2	107.4	C12—C11—H11	119.6
C8—C3—C4	118.3 (4)	C13—C12—C11	120.0 (4)
C8—C3—C2	118.6 (4)	C13—C12—H12	120.0
C4—C3—C2	123.1 (3)	C11—C12—H12	120.0
C3—C4—C5	121.1 (4)	C14—C13—C12	120.2 (4)
C3—C4—H4	119.5	C14—C13—H13	119.9
C5—C4—H4	119.5	C12—C13—H13	119.9
C6—C5—C4	119.8 (6)	C13—C14—C15	120.5 (4)
C6—C5—H5	120.1	C13—C14—H14	119.7
C4—C5—H5	120.1	C15—C14—H14	119.7
C7—C6—C5	119.0 (5)	C10—C15—C14	119.7 (3)
C7—C6—H6	120.5	C10—C15—H15	120.1
C5—C6—H6	120.5	C14—C15—H15	120.1
			
C9—N1—C2—C3	−120.7 (3)	C2—N1—C9—O1	2.0 (5)
C9—N1—C2—C1	114.0 (3)	C2—N1—C9—C10	−178.1 (3)
N1—C2—C3—C8	154.8 (3)	O1—C9—C10—C11	−33.8 (4)
C1—C2—C3—C8	−80.7 (4)	N1—C9—C10—C11	146.2 (3)
N1—C2—C3—C4	−27.3 (5)	O1—C9—C10—C15	145.7 (3)
C1—C2—C3—C4	97.1 (4)	N1—C9—C10—C15	−34.2 (4)
C8—C3—C4—C5	−0.6 (6)	C15—C10—C11—C12	1.5 (5)
C2—C3—C4—C5	−178.5 (4)	C9—C10—C11—C12	−179.0 (3)
C3—C4—C5—C6	−0.7 (8)	C10—C11—C12—C13	−2.4 (6)
C4—C5—C6—C7	1.8 (9)	C11—C12—C13—C14	1.7 (6)
C5—C6—C7—C8	−1.6 (10)	C12—C13—C14—C15	−0.1 (6)
C4—C3—C8—C7	0.8 (7)	C11—C10—C15—C14	0.2 (4)
C2—C3—C8—C7	178.8 (5)	C9—C10—C15—C14	−179.4 (3)
C6—C7—C8—C3	0.3 (9)	C13—C14—C15—C10	−0.9 (5)

### N-[(1S)-1-Phenylethyl]benzamide (I) Hydrogen-bond geometry (Å, º)

**Table:**

*D*—H···*A*	*D*—H	H···*A*	*D*···*A*	*D*—H···*A*
N1—H1···O1^i^	0.85 (3)	2.11 (3)	2.952 (3)	169 (3)

Symmetry code: (i) *x*+1, *y*, *z*.

### N-[(1S)-1-Phenylethyl]benzamide (II) Crystal data

**Table:**

C_15_H_15_NO	*F*(000) = 240
*M**_r_* = 225.28	*D*_x_ = 1.208 Mg m^−^^3^
Monoclinic, *P*2_1_	Ag *K*α radiation, λ = 0.56083 Å
*a* = 8.3496 (6) Å	Cell parameters from 15127 reflections
*b* = 5.2632 (2) Å	θ = 2.7–24.3°
*c* = 14.2969 (10) Å	µ = 0.05 mm^−^^1^
β = 99.800 (6)°	*T* = 295 K
*V* = 619.12 (7) Å^3^	Prism, colourless
*Z* = 2	0.34 × 0.19 × 0.14 mm

### N-[(1S)-1-Phenylethyl]benzamide (II) Data collection

**Table:**

Stoe Stadivari diffractometer	2456 independent reflections
Radiation source: Sealed X-ray tube, Axo Astix-f Microfocus source	1847 reflections with *I* > 2σ(*I*)
Graded multilayer mirror monochromator	*R*_int_ = 0.037
Detector resolution: 5.81 pixels mm^-1^	θ_max_ = 21.0°, θ_min_ = 2.7°
ω scans	*h* = −10→10
Absorption correction: multi-scan (X-AREA; Stoe & Cie, 2019)	*k* = −6→6
*T*_min_ = 0.574, *T*_max_ = 1.000	*l* = −18→18
14738 measured reflections	

### N-[(1S)-1-Phenylethyl]benzamide (II) Refinement

**Table:**

Refinement on *F*^2^	Secondary atom site location: difference Fourier map
Least-squares matrix: full	Hydrogen site location: mixed
*R*[*F*^2^ > 2σ(*F*^2^)] = 0.036	H atoms treated by a mixture of independent and constrained refinement
*wR*(*F*^2^) = 0.090	*w* = 1/[σ^2^(*F*_o_^2^) + (0.0529*P*)^2^] where *P* = (*F*_o_^2^ + 2*F*_c_^2^)/3
*S* = 0.94	(Δ/σ)_max_ < 0.001
2456 reflections	Δρ_max_ = 0.12 e Å^−^^3^
159 parameters	Δρ_min_ = −0.14 e Å^−^^3^
1 restraint	Extinction correction: SHELXL-2018/3 (Sheldrick 2015b), Fc^*^=kFc[1+0.001xFc^2^λ^3^/sin(2θ)]^-1/4^
0 constraints	Extinction coefficient: 0.076 (15)
Primary atom site location: dual	

### N-[(1S)-1-Phenylethyl]benzamide (II) Fractional atomic coordinates and isotropic or equivalent isotropic displacement parameters (Å^2^)

**Table:**

	*x*	*y*	*z*	*U*_iso_*/*U*_eq_	
O1	0.4368 (2)	0.7962 (3)	0.72214 (11)	0.0587 (5)	
N1	0.4801 (2)	0.3812 (3)	0.75328 (12)	0.0469 (5)	
H1	0.463 (3)	0.230 (5)	0.7361 (17)	0.056*	
C1	0.5445 (4)	0.2070 (5)	0.91253 (17)	0.0634 (7)	
H1A	0.431636	0.203287	0.918240	0.095*	
H1B	0.609341	0.231415	0.974081	0.095*	
H1C	0.573648	0.049095	0.886292	0.095*	
C2	0.5745 (3)	0.4245 (4)	0.84752 (14)	0.0477 (5)	
H2	0.533500	0.580228	0.872634	0.057*	
C3	0.7537 (3)	0.4647 (4)	0.84452 (15)	0.0513 (5)	
C4	0.8339 (4)	0.3259 (8)	0.7885 (2)	0.0915 (10)	
H4	0.777746	0.203331	0.749040	0.110*	
C5	0.9981 (4)	0.3612 (11)	0.7883 (2)	0.1148 (15)	
H5	1.050709	0.260491	0.749457	0.138*	
C6	1.0820 (4)	0.5382 (9)	0.8431 (2)	0.0947 (11)	
H6	1.191933	0.563011	0.841941	0.114*	
C7	1.0040 (4)	0.6813 (8)	0.9005 (4)	0.1196 (15)	
H7	1.060842	0.805082	0.939071	0.144*	
C8	0.8399 (4)	0.6433 (6)	0.9018 (3)	0.0898 (10)	
H8	0.787916	0.740396	0.941994	0.108*	
C9	0.4209 (2)	0.5733 (4)	0.69593 (14)	0.0417 (5)	
C10	0.3338 (2)	0.5054 (4)	0.59924 (14)	0.0395 (5)	
C11	0.2133 (3)	0.6681 (4)	0.55550 (16)	0.0487 (5)	
H11	0.186538	0.811620	0.587523	0.058*	
C12	0.1325 (3)	0.6186 (5)	0.46458 (17)	0.0572 (6)	
H12	0.050761	0.727543	0.436194	0.069*	
C13	0.1725 (3)	0.4090 (5)	0.41606 (16)	0.0528 (6)	
H13	0.118548	0.376985	0.354762	0.063*	
C14	0.2928 (3)	0.2463 (4)	0.45841 (15)	0.0501 (6)	
H14	0.320498	0.105115	0.425418	0.060*	
C15	0.3725 (3)	0.2925 (4)	0.54998 (14)	0.0463 (5)	
H15	0.452280	0.180724	0.578634	0.056*	

### N-[(1S)-1-Phenylethyl]benzamide (II) Atomic displacement parameters (Å^2^)

**Table:**

	*U*^11^	*U*^22^	*U*^33^	*U*^12^	*U*^13^	*U*^23^
O1	0.0812 (12)	0.0348 (7)	0.0555 (9)	0.0003 (7)	−0.0011 (8)	−0.0023 (7)
N1	0.0578 (11)	0.0363 (9)	0.0433 (10)	0.0003 (8)	−0.0009 (8)	0.0004 (8)
C1	0.0814 (17)	0.0588 (13)	0.0495 (14)	0.0020 (13)	0.0091 (12)	0.0091 (11)
C2	0.0601 (14)	0.0403 (10)	0.0406 (11)	0.0053 (9)	0.0031 (10)	−0.0026 (9)
C3	0.0588 (14)	0.0488 (12)	0.0419 (11)	0.0040 (10)	−0.0038 (10)	0.0036 (10)
C4	0.0678 (18)	0.134 (3)	0.0759 (18)	−0.018 (2)	0.0215 (15)	−0.049 (2)
C5	0.071 (2)	0.200 (5)	0.078 (2)	−0.015 (3)	0.0245 (16)	−0.046 (3)
C6	0.0579 (17)	0.131 (3)	0.091 (2)	−0.012 (2)	0.0007 (17)	0.013 (2)
C7	0.065 (2)	0.099 (2)	0.177 (4)	−0.0025 (19)	−0.030 (2)	−0.042 (3)
C8	0.0617 (17)	0.080 (2)	0.116 (2)	0.0118 (14)	−0.0177 (16)	−0.0407 (18)
C9	0.0412 (10)	0.0358 (9)	0.0483 (12)	0.0010 (8)	0.0082 (9)	0.0019 (9)
C10	0.0379 (10)	0.0370 (9)	0.0438 (11)	−0.0016 (8)	0.0074 (8)	0.0047 (8)
C11	0.0477 (12)	0.0392 (10)	0.0581 (13)	0.0030 (9)	0.0059 (10)	0.0015 (9)
C12	0.0530 (14)	0.0547 (14)	0.0592 (15)	0.0043 (10)	−0.0042 (11)	0.0078 (11)
C13	0.0527 (13)	0.0589 (13)	0.0452 (11)	−0.0118 (11)	0.0039 (10)	0.0040 (11)
C14	0.0518 (12)	0.0500 (13)	0.0503 (13)	−0.0043 (9)	0.0141 (10)	−0.0052 (10)
C15	0.0466 (12)	0.0415 (10)	0.0501 (12)	0.0034 (9)	0.0059 (10)	0.0025 (10)

### N-[(1S)-1-Phenylethyl]benzamide (II) Geometric parameters (Å, º)

**Table:**

O1—C9	1.232 (3)	C6—H6	0.9300
N1—C9	1.342 (3)	C7—C8	1.388 (5)
N1—C2	1.459 (3)	C7—H7	0.9300
N1—H1	0.84 (3)	C8—H8	0.9300
C1—C2	1.522 (3)	C9—C10	1.492 (3)
C1—H1A	0.9600	C10—C11	1.387 (3)
C1—H1B	0.9600	C10—C15	1.390 (3)
C1—H1C	0.9600	C11—C12	1.383 (3)
C2—C3	1.519 (3)	C11—H11	0.9300
C2—H2	0.9800	C12—C13	1.374 (4)
C3—C4	1.343 (4)	C12—H12	0.9300
C3—C8	1.368 (3)	C13—C14	1.379 (3)
C4—C5	1.385 (4)	C13—H13	0.9300
C4—H4	0.9300	C14—C15	1.386 (3)
C5—C6	1.336 (5)	C14—H14	0.9300
C5—H5	0.9300	C15—H15	0.9300
C6—C7	1.359 (5)		
			
C9—N1—C2	122.11 (18)	C6—C7—C8	120.3 (3)
C9—N1—H1	120.7 (17)	C6—C7—H7	119.9
C2—N1—H1	117.1 (17)	C8—C7—H7	119.9
C2—C1—H1A	109.5	C3—C8—C7	120.7 (3)
C2—C1—H1B	109.5	C3—C8—H8	119.6
H1A—C1—H1B	109.5	C7—C8—H8	119.6
C2—C1—H1C	109.5	O1—C9—N1	121.50 (19)
H1A—C1—H1C	109.5	O1—C9—C10	121.34 (18)
H1B—C1—H1C	109.5	N1—C9—C10	117.16 (17)
N1—C2—C3	112.09 (17)	C11—C10—C15	118.86 (19)
N1—C2—C1	109.10 (18)	C11—C10—C9	118.16 (18)
C3—C2—C1	112.80 (19)	C15—C10—C9	122.93 (18)
N1—C2—H2	107.5	C12—C11—C10	120.5 (2)
C3—C2—H2	107.5	C12—C11—H11	119.7
C1—C2—H2	107.5	C10—C11—H11	119.7
C4—C3—C8	117.7 (3)	C13—C12—C11	120.3 (2)
C4—C3—C2	122.3 (2)	C13—C12—H12	119.9
C8—C3—C2	119.9 (2)	C11—C12—H12	119.9
C3—C4—C5	121.5 (3)	C12—C13—C14	119.9 (2)
C3—C4—H4	119.2	C12—C13—H13	120.1
C5—C4—H4	119.2	C14—C13—H13	120.1
C6—C5—C4	120.8 (4)	C13—C14—C15	120.2 (2)
C6—C5—H5	119.6	C13—C14—H14	119.9
C4—C5—H5	119.6	C15—C14—H14	119.9
C5—C6—C7	118.9 (3)	C14—C15—C10	120.3 (2)
C5—C6—H6	120.6	C14—C15—H15	119.8
C7—C6—H6	120.6	C10—C15—H15	119.8
			
C9—N1—C2—C3	−85.6 (2)	C2—N1—C9—O1	−3.7 (3)
C9—N1—C2—C1	148.7 (2)	C2—N1—C9—C10	176.80 (18)
N1—C2—C3—C4	−41.1 (3)	O1—C9—C10—C11	−28.1 (3)
C1—C2—C3—C4	82.5 (3)	N1—C9—C10—C11	151.43 (19)
N1—C2—C3—C8	140.4 (2)	O1—C9—C10—C15	149.3 (2)
C1—C2—C3—C8	−95.9 (3)	N1—C9—C10—C15	−31.2 (3)
C8—C3—C4—C5	0.0 (5)	C15—C10—C11—C12	0.3 (3)
C2—C3—C4—C5	−178.5 (3)	C9—C10—C11—C12	177.8 (2)
C3—C4—C5—C6	−1.0 (6)	C10—C11—C12—C13	−0.9 (4)
C4—C5—C6—C7	1.0 (7)	C11—C12—C13—C14	0.5 (3)
C5—C6—C7—C8	0.0 (6)	C12—C13—C14—C15	0.5 (3)
C4—C3—C8—C7	1.0 (5)	C13—C14—C15—C10	−1.1 (3)
C2—C3—C8—C7	179.5 (3)	C11—C10—C15—C14	0.7 (3)
C6—C7—C8—C3	−1.0 (6)	C9—C10—C15—C14	−176.71 (19)

### N-[(1S)-1-Phenylethyl]benzamide (II) Hydrogen-bond geometry (Å, º)

**Table:**

*D*—H···*A*	*D*—H	H···*A*	*D*···*A*	*D*—H···*A*
N1—H1···O1^i^	0.84 (3)	2.30 (3)	3.123 (2)	168 (2)

Symmetry code: (i) *x*, *y*−1, *z*.

### N-[(1S)-1-Phenylethyl]benzamide (III) Crystal data

**Table:**

C_15_H_15_NO	*D*_x_ = 1.199 Mg m^−^^3^
*M**_r_* = 225.28	Ag *K*α radiation, λ = 0.56083 Å
Orthorhombic, *P*2_1_2_1_2_1_	Cell parameters from 15625 reflections
*a* = 5.2133 (3) Å	θ = 2.5–24.4°
*b* = 18.3625 (12) Å	µ = 0.05 mm^−^^1^
*c* = 26.0799 (19) Å	*T* = 295 K
*V* = 2496.6 (3) Å^3^	Prism, colourless
*Z* = 8	0.27 × 0.25 × 0.08 mm
*F*(000) = 960	

### N-[(1S)-1-Phenylethyl]benzamide (III) Data collection

**Table:**

Stoe Stadivari diffractometer	5439 independent reflections
Radiation source: Sealed X-ray tube, Axo Astix-f Microfocus source	2319 reflections with *I* > 2σ(*I*)
Graded multilayer mirror monochromator	*R*_int_ = 0.132
Detector resolution: 5.81 pixels mm^-1^	θ_max_ = 21.0°, θ_min_ = 2.6°
ω scans	*h* = −6→6
Absorption correction: multi-scan (X-AREA; Stoe & Cie, 2019)	*k* = −23→23
*T*_min_ = 0.361, *T*_max_ = 1.000	*l* = −33→33
46311 measured reflections	

### N-[(1S)-1-Phenylethyl]benzamide (III) Refinement

**Table:**

Refinement on *F*^2^	Secondary atom site location: difference Fourier map
Least-squares matrix: full	Hydrogen site location: mixed
*R*[*F*^2^ > 2σ(*F*^2^)] = 0.038	H atoms treated by a mixture of independent and constrained refinement
*wR*(*F*^2^) = 0.080	*w* = 1/[σ^2^(*F*_o_^2^) + (0.0282*P*)^2^] where *P* = (*F*_o_^2^ + 2*F*_c_^2^)/3
*S* = 0.74	(Δ/σ)_max_ < 0.001
5439 reflections	Δρ_max_ = 0.11 e Å^−^^3^
316 parameters	Δρ_min_ = −0.10 e Å^−^^3^
0 restraints	Extinction correction: SHELXL-2018/3 (Sheldrick 2015b), Fc^*^=kFc[1+0.001xFc^2^λ^3^/sin(2θ)]^-1/4^
0 constraints	Extinction coefficient: 0.0132 (13)
Primary atom site location: dual	

### N-[(1S)-1-Phenylethyl]benzamide (III) Fractional atomic coordinates and isotropic or equivalent isotropic displacement parameters (Å^2^)

**Table:**

	*x*	*y*	*z*	*U*_iso_*/*U*_eq_	
O1A	0.4387 (3)	0.69302 (12)	0.38661 (8)	0.0630 (6)	
N1A	0.0203 (5)	0.70377 (15)	0.36724 (11)	0.0568 (8)	
H1A	−0.136 (5)	0.6967 (16)	0.3772 (11)	0.068*	
C1A	−0.1440 (6)	0.70880 (18)	0.27970 (13)	0.0720 (10)	
H1AA	−0.143954	0.656603	0.277663	0.108*	
H1AB	−0.112684	0.728890	0.246299	0.108*	
H1AC	−0.307634	0.725268	0.291994	0.108*	
C2A	0.0663 (6)	0.73371 (17)	0.31654 (13)	0.0588 (9)	
H2A	0.229586	0.713993	0.304120	0.071*	
C3A	0.0901 (5)	0.81724 (18)	0.31768 (13)	0.0557 (9)	
C4A	−0.0701 (7)	0.8593 (2)	0.34610 (15)	0.0764 (11)	
H4A	−0.195949	0.837185	0.366024	0.092*	
C5A	−0.0493 (8)	0.9346 (2)	0.34597 (15)	0.0847 (12)	
H5A	−0.160183	0.962224	0.366004	0.102*	
C6A	0.1311 (7)	0.9684 (2)	0.31689 (16)	0.0800 (11)	
H6A	0.143867	1.018943	0.316643	0.096*	
C7A	0.2924 (7)	0.9271 (2)	0.28823 (16)	0.0868 (13)	
H7A	0.417606	0.949590	0.268377	0.104*	
C8A	0.2729 (6)	0.8514 (2)	0.28814 (14)	0.0772 (11)	
H8A	0.383777	0.823801	0.268047	0.093*	
C9A	0.2110 (6)	0.68623 (16)	0.39947 (13)	0.0526 (9)	
C10A	0.1348 (5)	0.65628 (17)	0.45033 (12)	0.0498 (8)	
C11A	0.2833 (6)	0.60179 (19)	0.47200 (15)	0.0702 (10)	
H11A	0.428210	0.584939	0.454929	0.084*	
C12A	0.2167 (7)	0.5726 (2)	0.51869 (16)	0.0845 (12)	
H12A	0.314346	0.535028	0.532549	0.101*	
C13A	0.0064 (7)	0.5985 (2)	0.54520 (14)	0.0759 (11)	
H13A	−0.036474	0.578974	0.576969	0.091*	
C14A	−0.1383 (6)	0.6530 (2)	0.52434 (15)	0.0712 (10)	
H14A	−0.279492	0.670892	0.542129	0.085*	
C15A	−0.0758 (6)	0.68183 (17)	0.47688 (14)	0.0620 (9)	
H15A	−0.176383	0.718594	0.462823	0.074*	
O1B	0.9295 (3)	0.77969 (11)	0.14990 (9)	0.0670 (7)	
N1B	0.5075 (5)	0.75516 (14)	0.14882 (12)	0.0616 (8)	
H1B	0.354 (5)	0.7718 (16)	0.1493 (12)	0.074*	
C1B	0.3496 (6)	0.63517 (17)	0.17383 (14)	0.0746 (11)	
H1BA	0.384355	0.646314	0.209113	0.112*	
H1BB	0.370027	0.583818	0.168261	0.112*	
H1BC	0.176985	0.649170	0.165628	0.112*	
C2B	0.5365 (6)	0.67688 (16)	0.13957 (14)	0.0601 (9)	
H2B	0.710627	0.663219	0.149944	0.072*	
C3B	0.5075 (6)	0.65798 (17)	0.08365 (14)	0.0558 (8)	
C4B	0.3156 (7)	0.6867 (2)	0.05320 (17)	0.0809 (12)	
H4B	0.201052	0.719768	0.067555	0.097*	
C5B	0.2882 (7)	0.6682 (2)	0.00274 (18)	0.0905 (13)	
H5B	0.156648	0.688641	−0.016579	0.109*	
C6B	0.4541 (8)	0.6197 (2)	−0.01948 (17)	0.0871 (12)	
H6B	0.436818	0.607068	−0.053830	0.105*	
C7B	0.6442 (8)	0.5905 (2)	0.00953 (19)	0.0933 (13)	
H7B	0.756746	0.557102	−0.005069	0.112*	
C8B	0.6727 (7)	0.60959 (19)	0.06030 (16)	0.0787 (11)	
H8B	0.806240	0.589440	0.079221	0.094*	
C9B	0.7071 (6)	0.80071 (17)	0.15121 (12)	0.0534 (8)	
C10B	0.6444 (6)	0.88026 (17)	0.15614 (13)	0.0548 (9)	
C11B	0.8059 (7)	0.92343 (19)	0.18443 (15)	0.0763 (11)	
H11B	0.948339	0.902977	0.200342	0.092*	
C12B	0.7580 (9)	0.9974 (2)	0.18947 (18)	0.0956 (14)	
H12B	0.864493	1.026153	0.209710	0.115*	
C13B	0.5537 (9)	1.0280 (2)	0.16455 (18)	0.0940 (15)	
H13B	0.523360	1.077740	0.167155	0.113*	
C14B	0.3948 (8)	0.9854 (2)	0.13589 (18)	0.0943 (14)	
H14B	0.256023	1.006218	0.118964	0.113*	
C15B	0.4386 (6)	0.91134 (19)	0.13184 (15)	0.0718 (11)	
H15B	0.328058	0.882535	0.112564	0.086*	

### N-[(1S)-1-Phenylethyl]benzamide (III) Atomic displacement parameters (Å^2^)

**Table:**

	*U*^11^	*U*^22^	*U*^33^	*U*^12^	*U*^13^	*U*^23^
O1A	0.0482 (11)	0.0706 (16)	0.0702 (17)	−0.0008 (11)	0.0031 (12)	0.0003 (13)
N1A	0.0446 (13)	0.0673 (19)	0.059 (2)	−0.0003 (15)	0.0043 (14)	0.0115 (16)
C1A	0.077 (2)	0.072 (2)	0.066 (3)	0.003 (2)	−0.005 (2)	0.002 (2)
C2A	0.0524 (18)	0.066 (2)	0.058 (2)	0.0073 (17)	0.0101 (19)	0.005 (2)
C3A	0.0477 (17)	0.063 (2)	0.056 (2)	0.0047 (17)	0.0012 (17)	0.0087 (19)
C4A	0.076 (2)	0.066 (3)	0.087 (3)	0.000 (2)	0.025 (2)	0.008 (2)
C5A	0.100 (3)	0.065 (3)	0.090 (3)	0.007 (2)	0.022 (3)	0.000 (2)
C6A	0.088 (2)	0.064 (3)	0.088 (3)	−0.010 (2)	−0.002 (3)	0.005 (2)
C7A	0.075 (2)	0.076 (3)	0.110 (4)	−0.008 (2)	0.012 (3)	0.026 (3)
C8A	0.069 (2)	0.077 (3)	0.085 (3)	0.003 (2)	0.019 (2)	0.014 (2)
C9A	0.0512 (17)	0.040 (2)	0.066 (3)	−0.0008 (16)	−0.0018 (18)	−0.0050 (17)
C10A	0.0493 (17)	0.049 (2)	0.051 (2)	−0.0044 (16)	−0.0011 (17)	−0.0017 (18)
C11A	0.067 (2)	0.076 (3)	0.068 (3)	0.012 (2)	0.001 (2)	0.009 (2)
C12A	0.092 (3)	0.084 (3)	0.078 (3)	0.018 (2)	−0.001 (3)	0.019 (2)
C13A	0.082 (2)	0.084 (3)	0.062 (3)	−0.013 (2)	−0.001 (2)	0.008 (2)
C14A	0.068 (2)	0.080 (3)	0.065 (3)	−0.003 (2)	0.004 (2)	−0.001 (2)
C15A	0.0582 (18)	0.059 (2)	0.068 (3)	0.0051 (17)	−0.004 (2)	0.008 (2)
O1B	0.0433 (11)	0.0619 (15)	0.096 (2)	0.0017 (11)	0.0019 (12)	−0.0003 (13)
N1B	0.0428 (13)	0.0458 (18)	0.096 (2)	0.0002 (14)	0.0010 (16)	−0.0044 (16)
C1B	0.076 (2)	0.055 (2)	0.093 (3)	−0.0052 (19)	0.002 (2)	0.006 (2)
C2B	0.0505 (17)	0.044 (2)	0.086 (3)	0.0020 (15)	−0.0057 (19)	−0.0008 (19)
C3B	0.0479 (17)	0.0464 (19)	0.073 (3)	−0.0015 (16)	0.0007 (19)	0.002 (2)
C4B	0.069 (2)	0.087 (3)	0.087 (3)	0.015 (2)	−0.007 (2)	−0.009 (3)
C5B	0.082 (3)	0.103 (3)	0.086 (4)	0.007 (3)	−0.010 (3)	0.006 (3)
C6B	0.085 (3)	0.103 (3)	0.073 (3)	−0.007 (3)	−0.005 (3)	−0.006 (3)
C7B	0.099 (3)	0.094 (3)	0.088 (4)	0.015 (3)	0.004 (3)	−0.013 (3)
C8B	0.075 (2)	0.076 (3)	0.085 (3)	0.015 (2)	−0.008 (2)	−0.004 (2)
C9B	0.0466 (16)	0.057 (2)	0.057 (2)	−0.0022 (17)	−0.0001 (16)	−0.0032 (18)
C10B	0.0516 (17)	0.049 (2)	0.064 (2)	−0.0046 (17)	0.0087 (18)	0.0032 (19)
C11B	0.070 (2)	0.062 (3)	0.097 (3)	−0.007 (2)	0.004 (2)	−0.009 (2)
C12B	0.105 (3)	0.061 (3)	0.121 (4)	−0.021 (2)	0.023 (3)	−0.015 (3)
C13B	0.111 (3)	0.049 (3)	0.122 (4)	0.002 (3)	0.040 (3)	0.011 (3)
C14B	0.090 (3)	0.068 (3)	0.125 (4)	0.009 (2)	0.012 (3)	0.030 (3)
C15B	0.068 (2)	0.056 (3)	0.091 (3)	0.0047 (19)	−0.001 (2)	0.011 (2)

### N-[(1S)-1-Phenylethyl]benzamide (III) Geometric parameters (Å, º)

**Table:**

O1A—C9A	1.240 (3)	O1B—C9B	1.223 (3)
N1A—C9A	1.341 (4)	N1B—C9B	1.336 (3)
N1A—C2A	1.452 (4)	N1B—C2B	1.465 (4)
N1A—H1A	0.87 (3)	N1B—H1B	0.85 (3)
C1A—C2A	1.528 (4)	C1B—C2B	1.528 (4)
C1A—H1AA	0.9600	C1B—H1BA	0.9600
C1A—H1AB	0.9600	C1B—H1BB	0.9600
C1A—H1AC	0.9600	C1B—H1BC	0.9600
C2A—C3A	1.539 (4)	C2B—C3B	1.507 (4)
C2A—H2A	0.9800	C2B—H2B	0.9800
C3A—C4A	1.357 (4)	C3B—C8B	1.379 (4)
C3A—C8A	1.376 (4)	C3B—C4B	1.382 (4)
C4A—C5A	1.387 (4)	C4B—C5B	1.367 (5)
C4A—H4A	0.9300	C4B—H4B	0.9300
C5A—C6A	1.359 (5)	C5B—C6B	1.370 (5)
C5A—H5A	0.9300	C5B—H5B	0.9300
C6A—C7A	1.357 (5)	C6B—C7B	1.357 (5)
C6A—H6A	0.9300	C6B—H6B	0.9300
C7A—C8A	1.395 (4)	C7B—C8B	1.378 (5)
C7A—H7A	0.9300	C7B—H7B	0.9300
C8A—H8A	0.9300	C8B—H8B	0.9300
C9A—C10A	1.490 (4)	C9B—C10B	1.502 (4)
C10A—C15A	1.380 (4)	C10B—C15B	1.370 (4)
C10A—C11A	1.386 (4)	C10B—C11B	1.372 (4)
C11A—C12A	1.375 (5)	C11B—C12B	1.388 (4)
C11A—H11A	0.9300	C11B—H11B	0.9300
C12A—C13A	1.381 (4)	C12B—C13B	1.368 (5)
C12A—H12A	0.9300	C12B—H12B	0.9300
C13A—C14A	1.367 (4)	C13B—C14B	1.363 (5)
C13A—H13A	0.9300	C13B—H13B	0.9300
C14A—C15A	1.385 (4)	C14B—C15B	1.382 (4)
C14A—H14A	0.9300	C14B—H14B	0.9300
C15A—H15A	0.9300	C15B—H15B	0.9300
			
C9A—N1A—C2A	122.6 (3)	C9B—N1B—C2B	122.8 (2)
C9A—N1A—H1A	118 (2)	C9B—N1B—H1B	120 (2)
C2A—N1A—H1A	119 (2)	C2B—N1B—H1B	117 (2)
C2A—C1A—H1AA	109.5	C2B—C1B—H1BA	109.5
C2A—C1A—H1AB	109.5	C2B—C1B—H1BB	109.5
H1AA—C1A—H1AB	109.5	H1BA—C1B—H1BB	109.5
C2A—C1A—H1AC	109.5	C2B—C1B—H1BC	109.5
H1AA—C1A—H1AC	109.5	H1BA—C1B—H1BC	109.5
H1AB—C1A—H1AC	109.5	H1BB—C1B—H1BC	109.5
N1A—C2A—C1A	109.9 (3)	N1B—C2B—C3B	112.0 (3)
N1A—C2A—C3A	111.9 (3)	N1B—C2B—C1B	109.3 (3)
C1A—C2A—C3A	111.6 (3)	C3B—C2B—C1B	112.7 (3)
N1A—C2A—H2A	107.7	N1B—C2B—H2B	107.5
C1A—C2A—H2A	107.7	C3B—C2B—H2B	107.5
C3A—C2A—H2A	107.7	C1B—C2B—H2B	107.5
C4A—C3A—C8A	118.2 (3)	C8B—C3B—C4B	116.4 (4)
C4A—C3A—C2A	121.8 (3)	C8B—C3B—C2B	120.9 (3)
C8A—C3A—C2A	119.9 (3)	C4B—C3B—C2B	122.7 (3)
C3A—C4A—C5A	121.2 (3)	C5B—C4B—C3B	122.3 (4)
C3A—C4A—H4A	119.4	C5B—C4B—H4B	118.9
C5A—C4A—H4A	119.4	C3B—C4B—H4B	118.9
C6A—C5A—C4A	120.8 (4)	C4B—C5B—C6B	120.2 (4)
C6A—C5A—H5A	119.6	C4B—C5B—H5B	119.9
C4A—C5A—H5A	119.6	C6B—C5B—H5B	119.9
C7A—C6A—C5A	118.7 (4)	C7B—C6B—C5B	118.8 (4)
C7A—C6A—H6A	120.6	C7B—C6B—H6B	120.6
C5A—C6A—H6A	120.6	C5B—C6B—H6B	120.6
C6A—C7A—C8A	120.9 (4)	C6B—C7B—C8B	120.9 (4)
C6A—C7A—H7A	119.6	C6B—C7B—H7B	119.5
C8A—C7A—H7A	119.6	C8B—C7B—H7B	119.5
C3A—C8A—C7A	120.2 (4)	C7B—C8B—C3B	121.4 (4)
C3A—C8A—H8A	119.9	C7B—C8B—H8B	119.3
C7A—C8A—H8A	119.9	C3B—C8B—H8B	119.3
O1A—C9A—N1A	121.1 (3)	O1B—C9B—N1B	122.6 (3)
O1A—C9A—C10A	122.2 (3)	O1B—C9B—C10B	121.0 (3)
N1A—C9A—C10A	116.7 (3)	N1B—C9B—C10B	116.3 (3)
C15A—C10A—C11A	119.0 (3)	C15B—C10B—C11B	119.2 (3)
C15A—C10A—C9A	122.2 (3)	C15B—C10B—C9B	122.4 (3)
C11A—C10A—C9A	118.7 (3)	C11B—C10B—C9B	118.3 (3)
C12A—C11A—C10A	120.1 (3)	C10B—C11B—C12B	120.4 (4)
C12A—C11A—H11A	119.9	C10B—C11B—H11B	119.8
C10A—C11A—H11A	119.9	C12B—C11B—H11B	119.8
C11A—C12A—C13A	120.6 (4)	C13B—C12B—C11B	119.8 (4)
C11A—C12A—H12A	119.7	C13B—C12B—H12B	120.1
C13A—C12A—H12A	119.7	C11B—C12B—H12B	120.1
C14A—C13A—C12A	119.4 (4)	C14B—C13B—C12B	119.9 (4)
C14A—C13A—H13A	120.3	C14B—C13B—H13B	120.0
C12A—C13A—H13A	120.3	C12B—C13B—H13B	120.0
C13A—C14A—C15A	120.4 (3)	C13B—C14B—C15B	120.4 (4)
C13A—C14A—H14A	119.8	C13B—C14B—H14B	119.8
C15A—C14A—H14A	119.8	C15B—C14B—H14B	119.8
C10A—C15A—C14A	120.4 (3)	C10B—C15B—C14B	120.2 (4)
C10A—C15A—H15A	119.8	C10B—C15B—H15B	119.9
C14A—C15A—H15A	119.8	C14B—C15B—H15B	119.9
			
C9A—N1A—C2A—C1A	147.9 (3)	C9B—N1B—C2B—C3B	−94.7 (4)
C9A—N1A—C2A—C3A	−87.5 (3)	C9B—N1B—C2B—C1B	139.6 (3)
N1A—C2A—C3A—C4A	−42.5 (4)	N1B—C2B—C3B—C8B	136.4 (3)
C1A—C2A—C3A—C4A	81.1 (4)	C1B—C2B—C3B—C8B	−99.8 (3)
N1A—C2A—C3A—C8A	139.4 (3)	N1B—C2B—C3B—C4B	−44.4 (4)
C1A—C2A—C3A—C8A	−97.0 (3)	C1B—C2B—C3B—C4B	79.3 (4)
C8A—C3A—C4A—C5A	−0.7 (5)	C8B—C3B—C4B—C5B	0.4 (5)
C2A—C3A—C4A—C5A	−178.8 (3)	C2B—C3B—C4B—C5B	−178.8 (3)
C3A—C4A—C5A—C6A	0.6 (6)	C3B—C4B—C5B—C6B	−0.1 (6)
C4A—C5A—C6A—C7A	−0.5 (6)	C4B—C5B—C6B—C7B	0.2 (6)
C5A—C6A—C7A—C8A	0.5 (6)	C5B—C6B—C7B—C8B	−0.7 (6)
C4A—C3A—C8A—C7A	0.7 (5)	C6B—C7B—C8B—C3B	1.1 (6)
C2A—C3A—C8A—C7A	178.8 (3)	C4B—C3B—C8B—C7B	−0.9 (5)
C6A—C7A—C8A—C3A	−0.6 (6)	C2B—C3B—C8B—C7B	178.3 (3)
C2A—N1A—C9A—O1A	−2.1 (5)	C2B—N1B—C9B—O1B	−5.9 (5)
C2A—N1A—C9A—C10A	179.8 (3)	C2B—N1B—C9B—C10B	174.6 (3)
O1A—C9A—C10A—C15A	144.0 (3)	O1B—C9B—C10B—C15B	143.9 (3)
N1A—C9A—C10A—C15A	−37.9 (4)	N1B—C9B—C10B—C15B	−36.6 (4)
O1A—C9A—C10A—C11A	−34.9 (4)	O1B—C9B—C10B—C11B	−33.5 (5)
N1A—C9A—C10A—C11A	143.2 (3)	N1B—C9B—C10B—C11B	146.0 (3)
C15A—C10A—C11A—C12A	1.6 (5)	C15B—C10B—C11B—C12B	1.6 (5)
C9A—C10A—C11A—C12A	−179.4 (3)	C9B—C10B—C11B—C12B	179.1 (3)
C10A—C11A—C12A—C13A	−1.9 (5)	C10B—C11B—C12B—C13B	−2.4 (6)
C11A—C12A—C13A—C14A	0.8 (5)	C11B—C12B—C13B—C14B	1.5 (7)
C12A—C13A—C14A—C15A	0.4 (5)	C12B—C13B—C14B—C15B	0.0 (6)
C11A—C10A—C15A—C14A	−0.4 (5)	C11B—C10B—C15B—C14B	0.0 (5)
C9A—C10A—C15A—C14A	−179.3 (3)	C9B—C10B—C15B—C14B	−177.4 (3)
C13A—C14A—C15A—C10A	−0.7 (5)	C13B—C14B—C15B—C10B	−0.8 (6)

### N-[(1S)-1-Phenylethyl]benzamide (III) Hydrogen-bond geometry (Å, º)

**Table:**

*D*—H···*A*	*D*—H	H···*A*	*D*···*A*	*D*—H···*A*
N1*A*—H1*A*···O1*A*^i^	0.87 (3)	2.23 (3)	3.080 (3)	167 (3)
N1*B*—H1*B*···O1*B*^i^	0.85 (3)	2.22 (3)	3.047 (3)	163 (3)

Symmetry code: (i) *x*−1, *y*, *z*.
